# Interleukin-6 targeting antibodies for the treatment of Myelin Oligodendrocyte Glycoprotein Antibody-associated Disease (MOGAD): A review of current literature

**DOI:** 10.3934/Neuroscience.2025008

**Published:** 2025-05-08

**Authors:** Siddarth R. Ganesh, Orion Yedidia, Krupa Pandey, Carmenrita Infortuna, Charitha Madiraju, Florian P. Thomas, Fortunato Battaglia

**Affiliations:** 1 Department of Medical Sciences, Hackensack Meridian School of Medicine, Nutley, New Jersey, USA; 2 Department of Neurology, Hackensack University Medical Center and Hackensack Meridian School of Medicine, Nutley, NJ, USA; 3 Department of Cognitive Sciences, Psychology, Educational and Cultural Studies, University of Messina, Messina, Italy

**Keywords:** MOGAD, Interleukin 6, tocilizumab, satralizumab, efficacy

## Abstract

Myelin oligodendrocyte glycoprotein antibody-associated disease (MOGAD) is an autoimmune inflammatory demyelinating disorder that can manifest as optic neuritis, transverse myelitis, and acute disseminated encephalomyelitis. Although typically monophasic, relapsing cases are more common in adults. Current treatments include corticosteroids, intravenous immunoglobulin, immune-suppressive drugs, and plasma exchange, but there is emerging interest in the use of interleukin-6 (IL-6) inhibitors to prevent relapses such as tocilizumab and satralizumab. This review analyzed 24 studies on IL-6 inhibitors for MOGAD, including case reports, case series, and retrospective studies with at least one MOGAD patient. Tocilizumab demonstrated significant efficacy, with most studies reporting reduced annualized relapse rates (ARR), prolonged relapse-free periods, and improved neurological outcomes, including stabilization or recovery of vision, motor function, and magnetic resonance imaging (MRI) lesion resolution. Satralizumab also showed potential, though data were more limited. While IL-6 inhibitors appear beneficial for steroid-dependent or treatment-resistant MOGAD, the existing data are limited to small, observational studies. Larger controlled trials are needed to establish their long-term efficacy and safety.

## 1. Introduction

Myelin oligodendrocyte glycoprotein antibody-associated disease (MOGAD) is an autoimmune disorder involving central nervous system demyelination with antibodies against myelin oligodendrocyte glycoprotein (MOG). Research conducted across various nations indicates that MOGAD is a worldwide condition impacting individuals of all age groups. The annual incidence of MOGAD ranges from 1.6 to 3.4 cases per million people, while its prevalence is estimated to be around 20 cases per million [1]–[3]. It presents with clinical features similar to other demyelinating diseases such as multiple sclerosis (MS) and aquaporin-4-IgG-positive neuromyelitis optica spectrum disorder (AQP4 + NMOSD), yet MOGAD is increasingly recognized as a separate clinical entity with a distinct pathophysiology and clinical management [4]. Optic neuritis is one of the most common symptoms, affecting about 50% of MOGAD patients, which tends to have a more favorable recovery than optic neuritis in MS [5],[6].

In more severe cases, transverse myelitis can cause paralysis and bladder dysfunction, although recovery tends to be better in MOGAD compared with AQP4 + NMOSD [7],[8]. Acute disseminated encephalomyelitis (ADEM) is more frequent in pediatric patients and is characterized by widespread inflammation in the brain and spinal cord, leading to fever, headache, confusion, and cognitive deficits, but recovery is generally favorable after the initial attack [5],[9],[10]. Despite its heterogeneous presentation, MOGAD is typically monophasic but it can be relapsing in some patients, with relapses being more common in adults with optic neuritis, often triggered by infections or vaccinations [5],[11]. The current treatment landscape for MOGAD primarily involves using corticosteroids, intravenous immunoglobulin (IVIG), and therapeutic plasma exchange to manage acute attacks and prevent future relapses [12]. More targeted medications, such as azathioprine, mycophenolate mofetil, and rituximab, have been used to reduce relapse rates [13]. In addition to the current treatment, Interleukin-6 (IL-6) is being investigated as a potential target for pharmacotherapies in the context of MOGAD [4]. In MOGAD's pathogenesis, IL-6 triggers key inflammatory mechanisms by impacting B lymphocyte maturation, transforming naive cells into antibody-producing plasma cells while accelerating mutation rates within germinal centers [14]. It upregulates MHC-II expression on antigen-presenting cells and activates STAT3 signaling in B cells, inducing Bcl-6 and Blimp-1 expression to create a transcriptional program favoring plasma cells' survival and sustained autoantibody production [15]. IL-6 blockade reduces circulating MOG-IgG titers and attenuates clinical disease severity in experimental models, suggesting its therapeutic potential [16]. Tocilizumab is an anti-IL-6 antibody approved for treatments of rheumatoid arthritis, cytokine release syndrome, giant cell arteritis, systemic juvenile idiopathic arthritis, and polyarticular juvenile idiopathic arthritis [17]–[19].

Another IL-6 antibody, satralizumab, is approved for the treatment of AQP4 + NMOSD [20]. The potential of IL-6 targeting antibodies in the treatment of MOGAD is still under investigation. For instance, the TANGO study demonstrated that tocilizumab outperforms azathioprine in preventing relapses and slowing disability progression in NMOSD patients. By reducing AQP4-IgG levels—a critical factor in NMOSD—tocilizumab proves effective across diverse patient groups, including those with additional autoimmune conditions [21]. While these results are encouraging, more research is needed to understand its long-term effects and applicability to a wider population. This systematic review aimed to consolidate current evidence on the therapies and assess their efficacy and limitations in improving patient outcomes.

## 2. Methods

**Figure 1.**
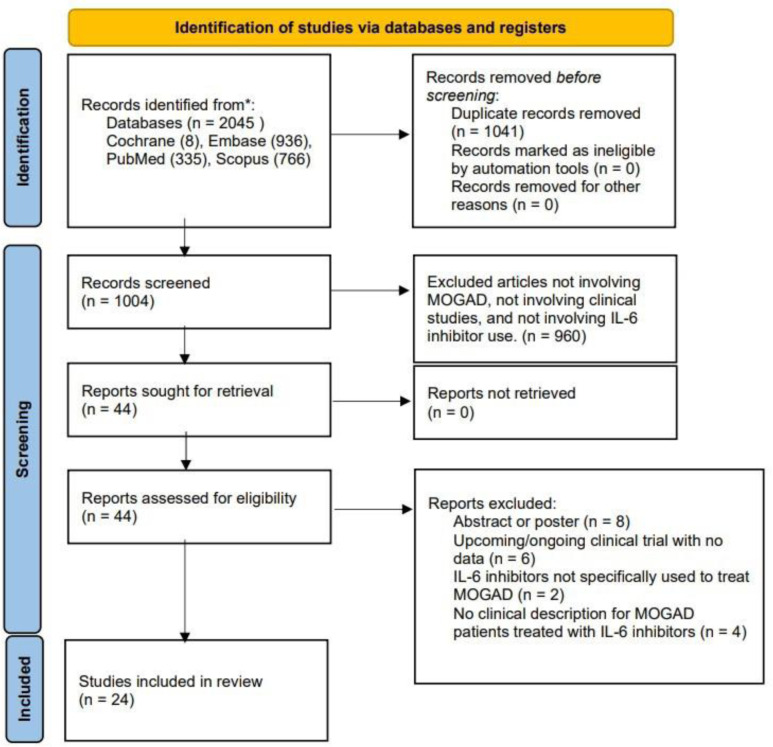
Flowchart of the study selection process based on PRISMA guidelines.

We conducted database searches for studies on 10 October 2024, which included Medline (through PubMed), Scopus, Embase, and Cochrane Library. We used keywords related to MOGAD as well as IL-6, which included variations of myelin oligodendrocyte glycoprotein, myelin oligodendrocyte glycoprotein-associated disease, Interleukin-6, anti-Interleukin-6, tocilizumab, and satralizumab. The search included the following keywords “MOG, myelin oligodendrocyte glycoprotein, myelin oligodendrocyte glycoprotein antibody-associated disease, MOGAD, MOG antibody disease, myelin oligodendrocyte glycoprotein antibody disorder, MOG antibody-associated disease, MOG antibody-associated disorder, Interleukin 6, Interleukin-6, IL-6, anti-Interleukin-6, anti-IL-6, tocilizumab, and satralizumab”. For all four databases, the words and phrases associated with MOGAD and IL-6 were separated by an “OR” statement. The two search strings were then combined with an “AND” statement, so that studies that mentioned both MOGAD variations as well as IL-6 variations could be obtained. For Scopus, the entire search string was preceded by “TITLE-ABS-KEY” so that the title, abstract, and keywords would be probed. For Embase, each keyword was followed by “/exp” so subjects related to them could also be searched for. The studies were restricted to the English language and published, full-text articles. We included studies with patients who have been diagnosed with MOGAD and who were treated with IL-6 inhibitors. Given the recent development of targeted therapies for the condition, we included all studies exploring the use of IL-6 inhibitors for MOGAD, including but not limited to case reports, cohort studies, case-control studies, and randomized controlled trials. Two reviewers (SG and OY) independently searched the databases for studies, collated them into a single collection, and selected which ones were to be included for analysis. Any discrepancies between the two reviewers were solved by a third reviewer (CM). The process is illustrated through a PRISMA flow diagram (Figure 1). Important parameters were obtained from each study, including the year of publication, the study design, the number of participants, the IL-6 inhibitor protocol, other therapies, laboratory findings, imaging findings, clinical efficacy, and adverse effects of tocilizumab (TCZ).

## 3. Results

A total of 24 studies are included in the review. Fifteen were case reports of patients with MOGAD treated with an IL-6 inhibitor, and one was a case series of patients with the disorder who had received at least one dose of an IL-6 inhibitor. These papers used tocilizumab as the IL-6 inhibitor and are included in Table 1. Eight other studies included at least one patient with MOGAD treated with an IL-6 inhibitor. One case series used satralizumab, another anti-IL-6 inhibitor, in the treatment protocol for MOGAD. We considered any patient with clinical findings and a positive MOG antibody serology as individuals with MOGAD.

**Table 1.** Summary of the parameters from studies.

Author (date)	Study design	Number of participants	IL-6 inhibitor protocol	Other therapies	Laboratory findings	Imaging findings (only CT/MRI, not including things like OCT)	Clinical efficacy	Adverse events (from TCZ)
Sbragia (2024) [22]	Case report	1	TCZ, 8 mg/kg intravenous (IV) monthly for 4 months, combined with prednisone at 25 mg daily	Initial therapy of high dose IV steroids followed by maintenance PO steroids at 50 mg/kg; RTX IV with PO steroids; high-dose IV steroids and 5 plasma exchange sessions; After TCZ, 2 cycles of IVIG infusion, hematopoietic stem cell transplant	Undetectable CD19+ B cells after RTX treatment	MRI: Infratentorial lesion, right ON atrophy, spinal demyelinating lesions. Further increase in the tumefactive spinal cord lesions after TCZ treatment. Infratentorial and spinal lesions reduced with HSCT	Continued radiological progression of MOGAD. EDSS from 3.0 to 6.0	
Masuccio (2020) [23]	Case report	1	TCZ 13 IV infusions per month (unspecified regimen)	Five 1000 mg RTX infusions IV	CD19+ cell depletion after RTX treatment		Paraparesis with 3/5 strength, EDSS 6.5, and maintenance of walking ability not altered during TCZ treatment and during COVID-19 infection	COVID-19 infection noted
McLendon (2023) [24]	Case report	2	TCZ, 2 doses 8 mg/kg/dose IV	4 days of MPS 1000 mg IV per day; 3 PLEX sessions or IVIG 2 g/kg, monthly IVIG for seizures	CSF lymphocytic pleocytosis, elevated intracranial pressure, normal protein/glucose; elevated serum IL-6 after an acute episode; ICPs normalized after TCZ; serum MOG antibody negative after treatment	MRI: mild leptomeningeal enhancement, cortical/subcortical hyperintensities, CT: cerebral edema	Glasgow Coma Scale improvement from 8 to 12 followed by full alertness, normal neurological examination with mild behavioral or cognitive impairment, monophasic normalized ICP for Patient 2, GCS 8 to 14	
Kwon (2024) [25]	Case report	1	TCZ, 8 mg/kg/dose IV, one acute dose, maintenance monthly for 2 years with IVIG 1 g/kg	MPS, IVIG, PO prednisone, RTX, PLEX, AZA	CSF: mildly elevated protein without pleocytosis	MRI: multiple bilateral cerebral nodular lesions, demyelinating lesions, mild brain atrophy	No relapse, no functional limitations	
Kang (2024) [26]	Case series	4	TCZ 8 mg/kg IV monthly (for 51.3, 33.6, 16.4, or 9.8 months)	MMF, RTX, IVIG; AZA, MMF, IVIG; MMF, IVIG; or IFN, MMF, IVIG. IVIG dosed at 0.4 g/kg every 4 weeks for all 4 patients	Negative oligoclonal bands		Relapse-free with a median follow-up of 25 months. EDSS remained the same from the initiation of TCZ to follow-up	
Elsbernd (2021) [27]	Case series	2	TCZ: 8 mg/kg monthly IV	Case 1: 5 days of IV MPS, prednisone taper, gabapentin, RTX (ultimately d/c due to treatment failure), resolved with TCZ Case 2: AZA with daily prednisone, RTX IVIG, prednisone		Case 1: longitudinally extensive transverse myelitis; patchy enhancement of the anterior, central and posterior cord; hyperintensity in the dorsal pons extending to the medulla; patchy enhancement of the right medial cerebellum and right medial subcortical white matter; Improvement in optic nerve enhancement after initiation of TCZ therapy Case 2: Hyperintensities in the diencephalon and corpus callosum, “H- sign” MOGAD myelitis with hyperintensity of the central gray matter	No relapses	Transient hyperlipidemia
Kim (2023) [28]	Case report	1	TCZ (unspecified regimen)	IVIG (2 g/kg for 5 days), delayed onset of response to RTX followed with TCZ	White blood cell (WBC) count = 250/µL, 90% lymphocytes, elevated proteins (127.6 mg/dL), slightly decreased glucose levels (54 mg/dL; 120 mg/dL in serum), and increased immunoglobulin G index (0.71). Oligoclonal bands were absent. The cytology was negative for malignant cells	MRI: Leptomeningeal enhancement that predominated in the left cerebral cortex; caudate nucleus lesion; whole-spine MRI was normal; 2 wk f/u MRI demonstrated improvement following TCZ	Improvement in fever, headache, and agraphia. Unspecified, but notable f/u MRI: CN lesions and LME were found to have improved in the follow-up brain MRI performed 2 weeks after the initial MRI	
Virupakshaiah (2024) [29]	Case report	1	TCZ (8 mg/kg IV dose every 4 weeks), slow oral prednisone taper 10 months later	IV MPS 1,000 mg daily for 3 days followed by oral prednisone at 60 mg daily, 5 sessions of PLEX, with anticoagulation	Red blood cell (RBC) count of 105 cells/µL, WBC count of 87 cells/µL (85% polys), and 89 mg/dL protein; serum MOG-IgG was positive at a titer level of 1:1,000; CSF Interleukin-6 (IL-6) level was elevated at 17,904.6 pg/mL (normal level ≤7.5 pg/mL)	MRI: Bilateral supratentorial lesions associated with venous sinus thrombosis, hemorrhage, and midline shift; small T2-FLAIR hyperintense lesions of the right frontal subcortical white matter and right lateral pons with mild enhancement. Thoracic spine MRI demonstrated T2 hyperintensity at T2–3 and enhancement involving the ventral cord at T2–3 and T6–7; T2-FLAIR hyperintense lesions involving the white matter and left globus pallidus with extensive hemorrhage and peripheral enhancement. Bilateral thalamic T2-FLAIR hyperintensity with hemorrhage and bilateral transverse sinus thrombosis	No known relapse. Ambulated without assistance but struggled with neurocognitive deficits, has not been able to return to work	
Lee (2023) [30]	Case report	1	TCZ (4 mg/kg IV dose for 1day; 2 doses given every 2 weeks, then doses given once a month) given concurrently with RTX (375 mg/m^2^ at regular intervals; the first 4 doses were administered weekly, with monthly dosing thereafter)	Intravenous acyclovir (d/c after negative viral polymerase chain reaction (PCR) results) and IVIG (0.4 g/kg for 5 days), RTX (375 mg/m^2^ at regular intervals; first 4 doses were administered weekly, with monthly dosing thereafter) on hospital Day 9 and TCZ on hospital Day 10; risperidone at 4 mg/day, and haloperidol at 5 mg together with lorazepam at 4 mg were given when needed during acute period of severe psychotic symptoms; risperidone at 1 mg/day was used as a maintenance treatment after the acute period	Pleocytosis (white blood cell count, 33/mm^3^, lymphocyte-predominant; red blood cell count, 280/mm^3^; protein concentration, 41 mg/dL; CSF glucose concentration, 59 mg/dL serum concentration, 101 mg/dL) and viral PCR negative; aquaporin-4 antibody result negative; MOG antibody result positive; CSF oligoclonal band positive	MRI: Small nonspecific T2 hyperintensities in bilateral cerebral white matter and no abnormal focal enhancing lesions; computed tomography imaging on the 38th day after hospitalization showed relative and mild cerebral perfusion asymmetry at the left fronto-parieto-temporal cortex	Most symptoms improved by 2 weeks following discharge; however, intermittent auditory hallucination misperceptions of TV sounds and autotopagnosia of the left hemibody remained	
Nagahata (2022) [31]	Case report	1	TCZ IV (400 mg/week for four weeks), followed by high-dose prednisolone	Prednisolone (60 mg/day) and methotrexate (10 mg/week), followed by IV MPS at a dose of 1 g/day for three days; RTX (500 mg/week for four weeks)	White blood cell count of 10,800/µL, hemoglobin level of 12 g/dL, platelet count of 300,000/µL, C-reactive protein level of 4.2 mg/dL, and no electrolyte, liver, or renal function test abnormalities. The level of myeloperoxidase–antineutrophil cytoplasmic antibody (MPO-ANCA) was elevated (27 IU/mL, normal levels are less than 3.5 IU/mL). The test results for antinuclear antibodyies anti- SSA/SS-B antibodies, and PR3-ANCA were negative, as were the *Treponema pallidum* hemagglutination and rapid plasma reagin tests; cavernous sinusitis and a low contrast-enhanced right optic nerve sheath	MRI: Cavernous sinusitis and a low contrast-enhanced right optic nerve sheath with a residual contrast effect around the right optic nerve	Visual acuity and field recovered to the pre-onset level, no recurrence of vision loss, and the prednisolone dose was tapered to 5 mg/day over the next 18 months with TCZ	
Smoot (2021) [32]	Case report	1	TCZ at 4 mg/kg IV monthly infusions	MPS at 1000 mg IV for 5 days, prednisone at 20 mg PO per day for 1 month	Elevated MOG titer before TCZ	Initial MRI: Nonspecific white matter changes, no intrinsic spinal cord signal; MRI after attack: Lesion from pons to cervical cord, C3–C5 lesion; MRI after treatment: reduction in brainstem and cervical cord lesions	No new relapse	
Kroenke (2022) [33]	Case report	1	TCZ IV (8 mg/kg) monthly alternating with IVIG (1 g/kg); switched to only IVIG (1 g/kg) after 4 months; restarted monthly TCZ (8 mg/kg) and IVIG (1 g/kg) infusions after 7 months; switched to only TCZ (8 mg/kg) monthly; restarted monthly TCZ (8 mg/kg) and IVIG (1 g/kg)	Initial: high-dose MPS IV at 1000 mg daily for 5 days and IVIG 2 g/kg. Second admission: High-dose MPS IV and 2 g/kg of IVIG over 3 days, 2 doses of RTX. Third admission: High-dose MPS IV and 2 g/kg of IVIG. Monthly IVIG (1 g/kg) and MPS IV (1000 mg) infusions every two weeks for 4 months	CSF: Elevated protein. Negative infectious workup. B cell suppression after RTX treatment	Initial MRI: Evidence of ADEM; third MRI: increasing ADEM and bilateral optic neuritis. Fourth MRI: New T2 lesions. Follow-up MRI with monthly IVIG and IVMP: Large lesion in the left superior and middle frontal gyrus. MRI after initial TCZ: No new lesions. Follow-up MRI with only IVIG: Large left hemisphere lesion. MRI after TCZ restarted: Reduction in the lesion. MRI with only TCZ: New lesions	Asymptomatic with large MRI lesions after TCZ's removal from combined treatment. Increased drowsiness and confusion with new MRI lesions after IVIG's removal from combined treatment	
Novi (2019) [34]	Case report	1	TCZ at 8 mg/kg IV every 4 weeks (24 months and ongoing)	Prednisone at 50 mg/day PO for 1 month, MPS at 1 g/day IV. RTX at 1 g IV twice; 2 more MPS IV doses	CSF: Normal cell count and protein. B cell depletion after RTX. Persistence of high MOG-antibody titers after TCZ.	Initial MRI: Negative. Second MRI: T4–T6, T8–T10, and T12–L1 lesions. Third MRI: New lesions in C5 and T5. MRI after TCZ: Reduction in cervical and thoracic spine lesions	No new relapses	
Schirò (2024) [35]	Case report	1	TCZ at 8 mg/kg IV every 4 weeks (February 2022–October 2023); subcutaneous TCZ at 162 mg every 2 weeks (6 total doses)	IFN-beta-1a, then natalizumab after relapse, then steroid therapy and PLEX, then RTX at 1000 mg every 2 weeks with leukoencephalopathy, then PLEX	Absence of oligoclonal bands in CSF, 15 pg/mL IL-6 before TCZ	Initial MRI: bilateral demyelinating lesions of frontal, temporal, parietal white matter; lesions in the cerebellum, midbrain, and medulla. Second MRI: Widespread leukoencephalopathy. MRI during TCZ treatment: Lesion reduction in the middle cerebellar peduncle and right mid-superior frontal gyrus. MRI after subcutaneous doses: No new lesions	No new clinical events, moderate recovery of cognitive function and walking capacity	COVID-19 during IV and subcutaneous regimen; *Pseudomon as aeruginosa* infection due to port-a-cath placement
Hayward-Koennecke (2019) [36]	Case report	1	TCZ at 8 mg/kg IV for 12 months and then tapered to every 6 weeks. Bimonthly treatments afterwards	Initial therapy: High-dose steroids. Six relapses treated with high-dose steroids. Then three natalizumab and two 1 g RTX doses. Then 12 doses of cyclophosphamide	CSF: 15 mononuclear cells/µL, elevated protein	Initial MRI: Lesions in all 3 spinal cord levels, bilateral pons, and bilateral thalamus. MRI after TCZ: No new lesions	No new relapses	
Dayrit (2024) [37]	Case report	1	TCZ at 320 mg IV every month for 5 months	Two sessions of MPS at 1 g IV every 24 hours for 5 days; four sessions of complete RTX at 500 mg IV after desensitization and after TCZ		MRI: Lesion in the central intramedullary region at the C2–C3 level	One relapse noted likely due to SARS-CoV-2 infection	Alopecia
Bilodeau (2024) [38]	Retrospective cohort	6	TCZ given for a total of 7.59 patient-years (unspecified dosage)				Four patients were reported to be relapse-free; however, one patient experienced 2 relapses due to highly refractory disease	
Lotan (2020) [39]	Retrospective cohort	2	TCZ, subcutaneous injection (162 mg for 1–2 weeks); one patient cotreated with MMF	RTX or azathropine	Two patients became MOG antibody seronegative after TCZ	MRI: No new lesions	No relapse	Hyperchole sterolemia
Rempe (2024) [40]	Retrospective cohort	1	TCZ (unspecified dosage)	Prior RTX, IVIG, MMF, MTX, and CYC			Relapse-free	
Ringelstein (2022) [41]	Retrospective cohort	14	TCZ at a median dose of 8.0 mg/kg IV (for MOGAD, NMOSD, and double seronegative); median treatment duration of 16.3 months for MOGAD. Two had IV MPS cotreatment; one had IV MPS and IVIG co-treatment; 1 had methotrexate co-treatment; one replaced TCZ with RTX	RTX, Azathioprine, MMF, low-dose steroid monotherapy, cyclophosphamide, IVIG, plasma exchange, and belimumab	Two showed decreased MOG titers	Reduction in lesions after treatment	ARR decrease (1.75 to 0), 79% relapse free. Three had relapses. EDSS 2.75 to 2.0	Infusion reaction, recurrent urinary tract infection, viral respiratory infections, pyelonephritis, neutropenia, liver enzyme change, mild cholesterol (low-density lipoprotein and high-density lipoprotein elevation)
Guzmán (2023) [42]	Case series	1	Satralizumab subcutaneously (unspecified dosage) and mycophenolate	RTX and mycophenolate. Prednisone PO low dose (either continuous or after satralizumab)	CD19 counts of 0% after RTX treatment	b/l optic neuritis, multifocal transverse myelitis, brainstem encephalitis	No new relapses. No improvement in visual acuity	
Rigal (2020) [43]	Case series	3	TCZ (8 mg/kg for monthly intravenous cycles and 162 mg weekly for subcutaneous injections)	RTX before TCZ		MRI stable following TCZ	Relapse-free	Hypertriglyceridemia, tooth infection
Powers (2020) [44]	Case series	1	Case 1: Monthly unspecified dosage	Case 1: RTX infusions every 6 months as well as monthly methylprednisone and IVIG infusions, clomipramine, quetiapine Case 2: Steroid trial, ketorolac, MPS, and valproate, vancomycin, ceftriaxone, acyclovir, lorazepam, fosphenytoin, and levetiracetam for status epilepticus development, 3 days of IV methylprednisone and 2 days of IVIG, RTX along with monthly IVIG and steroid infusions Case 3: IV steroids followed by an oral steroid taper	Case 1: MOG titer reduced after TCZ, not RTX Case 2: Elevated erythrocyte sedimentation rate, a normal C-reactive protein, positive (1:640, speckled pattern), and a negative serum neuromyelitis optica/AQP4 IgG-antibody Case 3: MOG-antibody seropositivity (titer 1:40)	MRIs Case 2: Cortical restricted diffusion with an area of increased T2 signal involving the left inferior posterior temporal lobe of the left frontal lobe Case 3: Expansile T2 hyperintensity in the central cord from C4 to C6	Return to baseline health (TCZ treatment was only noted in Case 1); Cases 2 and 3 resolved with steroid treatment	
Jelcic (2019) [45]	Case report	1	TCZ at 8 mg/kg IV per month	High-dose corticosteroids, then natalizumab at 300 mg monthly for 2 months, then high-dose corticosteroids and plasma exchange. RTX at 375 mg/m^2^ and 1000 mg separated by 1 month. Monthly cyclophosphamide at 600–1100 mg/m^2^/cycle for 13 months	Negative for infection	Initial MRI: Bilateral thalamic, mesencephalic, pontine, cervical, thoracic and lumbar spinal lesions MRI after all treatments: Right optic nerve atrophy, multiple supratentorial lesions, resolution of spinal lesions	No relapse for 17 months, no further deterioration of vision	

Note: TCZ, tocilizumab; RTX, rituximab; OCT, optical coherence tomography; HSCT, autologous hematopoietic stem cell transplantation; EDSS, expanded disability status scale; MPS, methylprednisolone; MOG, myelin oligodendrocyte glycoprotein; IVIG, intravenous immunoglobulin; PLEX, plasmapheresis; CSF, cerebrospinal fluid; AZA, azathioprine; IFN, interferon; MMF, mycophenolate mofetil; CN, cranial nerve; LME, leptomeningeal enhancement; FLAIR, fluid-attenuated inversion recovery; PO, per os; ARR, annualized relapse rate.

The dosage of tocilizumab was relatively similar across studies. Generally, 8 mg/kg IV was given, with some instances of 4 mg/kg [30],[32]. The drug was sometimes given concurrently with other treatments, such as PO prednisone, IV rituximab, and IVIG [22],[25],[31],[33]. Some regimens included treatment after tocilizumab, including PO prednisone, high-dose prednisolone alternating with IVIG, and subcutaneous tocilizumab [29],[31],[33],[35]. All studies included patients who have continued to have attacks while receiving other immunosuppressive therapies, including rituximab, IVIG, plasma exchange, methylprednisolone, prednisone, azathioprine, mycophenolate mofetil, methotrexate, interferon-beta-1a, natalizumab, acyclovir, and cyclophosphamide [46]. Patients who had previously received rituximab had low or undetectable CD19+cell populations but continued to have attacks [22],[23],[34]. Serum MOG antibodies vary before and after tocilizumab treatment. In one case, the patient became MOG antibody seronegative after treatment with tocilizumab, plasma exchange, and IVIG [24]. However, some patients continued to have high MOG antibody titers after treatment [29],[34]. Other studies demonstrated a decrease in MOG antibody titers or a conversion to seronegative status after treatment [39]–[41],[44].

For most of the patients, the tocilizumab prevented further relapses. In one case series, patients on monthly IV tocilizumab were relapse-free for a median of 25 months [26]. Some patients required additional treatment due to continued relapses. One patient with a severe relapse required IVIG and a hematopoietic stem cell transplant after tocilizumab use to stop relapses [22]. Another patient relapsed in the setting of SARS-CoV-2 infection, a third patient had two relapses [37],[38]. Most studies (Table 1) reported an improvement in or maintenance of clinical status after tocilizumab. For example, the neurological status of two pediatric patients who had severe MOGAD attacks improved neurologically as measured by the Glasgow Coma Scale from 8 to 12 or 14 after treatment [24]. The expanded disability status scale (EDSS) results were not altered with the initiation of tocilizumab for two patients but worsened from 3 to 6 for one individual with a severely refractory disease [22],[23],[26]. Tocilizumab administration resulted in improved headache, visual acuity, cognitive function, and gait [28],[31],[47]. In some patients, cognitive deficits from MOGAD attacks persisted after tocilizumab [29],[30]. One patient required alternating tocilizumab and IVIG; discontinuation of IVIG resulted in increased confusion and drowsiness [33]. In a retrospective NMOSD cohort study with some MOGAD patients (*n* = 14), tocilizumab reduced the annual relapse rate from 1.75 to 0 (*p* = 0.0011), with 79% of patients remaining relapse-free, with a reduction in EDSS from 2.5 to 2.0 (*p* < 0.031) [41].

MRI findings demonstrated improvements in demyelinating lesions after tocilizumab. Common MRI abnormalities in MOGAD include optic nerve, cerebral, thalamic, multiple-level spinal cord, brainstem, and leptomeningeal enhancements [22],[24],[25],[36],[37],[45]. Tocilizumab improved optic nerve and leptomeningeal enhancement, as well as lesion load in the caudate nucleus, brainstem, cervical spine, thoracic spine, and frontal gyrus [27],[28],[32],[34],[47]. The treatment was associated with the absence of new lesions or lesion progression in two cases [36],[47]. In one case, tumefactive spinal cord lesions continued to progress with tocilizumab [22]. In another case, the removal of tocilizumab or IVIG from the regimen resulted in new MRI lesions [33]. The key clinical findings are summarized in Table 2.

**Table 2.** Overall clinical characteristics from studies. EDSS, expanded disability status scale.

**Characteristics**	**IL-6 inhibitor therapy (*n* = 50)**
Patient type	33 adults
	10 pediatrics
	7 unknown
Time to next relapse	41 no new relapses
	4 between 4 and 12 months
	5 relapses with unknown time
Change in EDSS	14 trending down
	1 trending up
	5 no change
	30 unknown
Radiological improvement	16 improved
	9 no improvement
	25 no information

No adverse events were reported in most studies included in the review. One patient who exhibited a hypersensitivity reaction to rituximab experienced alopecia after tocilizumab, requiring desensitization before subsequent readministration of rituximab [37]. Reported adverse events in studies involving NMOSD, MOG antibody-positive patients included an infusion reaction (*n* = 1), high cholesterol (*n* = 1), hypertriglyceridemia (*n* = 1), and tooth infections (*n* =1) [39],[41],[43].

## 4. Discussion

Most studies included in this review demonstrate the effectiveness of tocilizumab in treating MOGAD. Most patients treated with tocilizumab experienced reductions in relapse frequency, with many remaining relapse-free for extended periods. Improvements in neurological symptoms and MRI lesions were common, suggesting both symptomatic and structural benefits from IL-6 inhibition. Tocilizumab's success in preventing relapses and improving clinical outcomes in MOGAD is notable, particularly for patients with refractory diseases who had failed other immunosuppressive regimens. The reduction in headache with improvements in gait, visual symptoms, and MRI lesions, along with a modest improvement in cognition, indicate that IL-6 inhibitors may confer broad neurological benefits.

Currently, only case reports and series support the use of IL-6 inhibitors. There are some retrospective cohort studies that include some MOGAD patients, but they either include other immunosuppressive medications or patients with AQP4 + NMOSD, with a limited number of MOG antibody-positive individuals treated with IL-6 inhibitors [38],[39],[41]. The short follow-up duration in many of these reports limits our understanding of tocilizumab's long-term efficacy and safety. Variations in previous concurrent therapies further complicate the interpretation of tocilizumab's isolated effects. While many studies used tocilizumab as the drug of choice for IL-6 inhibition, satralizumab was used in one patient for MOGAD, which resulted in no new relapses [42].

The effectiveness of tocilizumab in treating MOGAD implies the importance of IL-6 signaling in pathogenesis and disease progression. IL-6 is a pleiotropic cytokine with significant roles in regulating immune responses, inflammation, and hematopoiesis via multiple mechanisms such as macrophage differentiation and T cell survival [48]. The cytokine is often upregulated in chronic autoimmune diseases such as rheumatoid arthritis, inflammatory bowel disease, and systemic lupus erythematosus [49],[50]. IL-6 plays a significant role in the pathogenesis of central nervous system (CNS) inflammatory demyelinating diseases. For instance, IL-6 is increased in multiple sclerosis (MS) patients with an acute relapse, and it is implicated in T cell dysfunction [51],[52]. In NMOSD, IL-6 has been shown to promote plasmablast survival, disrupt the blood–brain barrier's integrity, enhance proinflammatory T-lymphocyte differentiation and activation, and stimulate the production of anti-aquaporin-4 antibodies [53],[54]. Moreover, IL-6 has been shown to correlate with disease activity and severity, with patients having higher levels of IL-6 during a relapse [55]. In addition, a negative correlation between IL-6 levels and brain volume has been observed during relapse and remission [55].

Concerning the pathophysiology of MOGAD, IL-6 is a pivotal driver in CNS inflammation and autoimmunity. Released by the innate immune cells, particularly the macrophages at inflammation sites, IL-6 promotes the differentiation of naive T cells into pro-inflammatory TH17 cells, while inhibiting regulatory T cell (Treg) formation, thus fostering a TH17-dominant environment. IL-6 also plays a role in activating and maturing B cells into plasma cells, which secrete autoantibodies. Pathogenic mechanisms in MOGAD involve interactions among IL-6, TH17.

CD4+ T cells, macrophages, and plasma B cells, contributing to an inflammatory and demyelinating pathology. Along with granulocytes, macrophages/microglia, and an activated complement, CD4+ T cells are dominant in MOGAD lesions, unlike in MS, where CD8+ T cells predominate [56],[57]. In addition, CD4+ Tregs are elevated in nonrelapsing MOGAD (MOGNR), but they are decreased in relapsing MOGAD (MOGR), further supporting the role of CD4+ T cells in the pathophysiology of MOGAD. This response from Tregs to rhMOG in MOGR indicates a likely loss of tolerance to the MOG autoantigen in MOGR [58]. MOGAD's pathogenesis appears to be predominantly T cell-driven. MOG-specific T cells are required to initiate CNS inflammation in experimental autoimmune encephalomyelitis (EAE), a key model for autoimmune neuroinflammation. In contrast, MOG-specific antibodies produced by B cells, while capable of exacerbating inflammation and demyelination, are neither necessary nor sufficient to cause disease. This suggests that cellular immunity, particularly T cell-mediated processes, plays a central role in MOGAD, with humoral immunity likely serving a secondary, amplifying role [57],[59]. Clinically, targeting B cells to relieve MOGAD attacks has produced variable responses. Rituximab results in an approximately 33% relapse-free rate after a 2-year follow-up, and a meta-analysis demonstrated that the drug results in 55% of patients not relapsing [60],[61]. In the studies selected for this review, patients who received rituximab continued to have clinical symptoms even after B cell depletion, which suggests that this cell type has less of a role in disease severity or relapse; 14 out of the 21 patients received rituximab but were subsequently treated with tocilizumab due to treatment failure. By inhibiting IL-6 signaling, tocilizumab seems to reduce the activation of pro-inflammatory T cells and B cells, thereby improving outcomes in severe or relapsing cases of MOGAD. MOG antibody levels in patients included in the review were variable, with some becoming seronegative after tocilizumab treatment and others maintaining high titers. Further data may be needed to establish a link between MOG antibody levels and clinical outcomes after treatment with IL-6-depleting therapies.

On the whole, tocilizumab shows promise in reducing relapses and stabilizing disease in refractory MOGAD, particularly in patients who are unresponsive to corticosteroids, IVIG, or rituximab, as evidenced by the case series and small cohort studies included in this review. Its IL-6 blockade may suppress pathogenic inflammation, with reports of sustained remission and steroid-sparing effects. Additional studies are necessary to solidify the role of IL-6 inhibitors in MOGAD, especially more extensive cohort studies and randomized controlled trials that can more definitively evaluate tocilizumab's efficacy and safety. Notably, tocilizumab demonstrates differential effects depending on the disease stage in MOGAD. Its rapid suppression of IL-6-mediated inflammation during acute attacks can reduce blood–brain barrier disruption and clinical severity, particularly in steroid-refractory cases [62]. In contrast, during the convalescent phase, its role appears more preventive, with sustained treatment leading to fewer relapses and slower disability progression [41]. However, heterogeneity in study designs limits definitive conclusions, warranting further prospective trials. Two clinical trials have been initiated to assess the efficacy and safety of IL-6 inhibitors in MOGAD. The TOMATO study is a randomized controlled multi-center Phase 2/3 study aiming to assess the efficacy and safety of tocilizumab with prednisone as a co-treatment for MOGAD [63]. The METEOROID study is a randomized controlled multi-center Phase 3 study that evaluates the efficacy and safety of satralizumab for MOGAD [64]. Both studies are currently in the recruiting phase, with an expected completion date in 2026. This review highlights the limitations of this therapeutic option. Data indicate that clinical outcomes fluctuate markedly among individuals [26], reflecting disease mechanisms that extend beyond simple IL-6 blockade [41]. The weeks-long delay before therapeutic benefits emerge creates real management dilemmas when rapid symptom control is essential [41]. Treatment risks reinforce this complexity, such as heightened infection vulnerability in mobility-impaired patients and documented cases where neurological function unexpectedly worsens following administration [47]. Furthermore, our review emphasizes that while tocilizumab is effective in MOGAD, some patients respond poorly due to factors like delayed treatment (linked to accumulated damage) [21], severe baseline disease (frequent relapses, high steroid use, EDSS ≥ 4) [41], and incomplete IL-6 blockade (persistent intrathecal inflammation, elevated CSF IL-6) [54]. Comorbid autoimmunity, particularly AQP4-IgG co-positivity or systemic autoimmune features, may further diminish the treatment response [65]. We should emphasize that while tocilizumab shows promise in neuroinflammatory disorders, its safety profile in neurology warrants caution due to risks of infections (including opportunistic and viral reactivation), hepatotoxicity, gastrointestinal perforation, and dyslipidemia, potentially exacerbating cerebrovascular disease. Neurology-specific concerns include breakthrough inflammation and seizure exacerbation (though rare), necessitating rigorous pre-treatment screening (e.g., hepatitis B virus, tuberculosis, liver function) and ongoing monitoring [Complete Blood Count (CBC), lipids, infection surveillance]. TCZ should be used judiciously in high-risk patients, particularly those on concurrent immunosuppressants or with comorbidities, balancing efficacy against these safety challenges.

## 5. Conclusions

In this review, the available evidence on tocilizumab as a treatment for MOGAD remains limited. Preliminary data suggest potential benefits in reducing relapse rates and improving outcomes in refractory cases. Without reliable markers predicting treatment success and facing the prohibitive costs restricting patient access, clinicians must approach IL-6 inhibition with measured expectations pending more definitive research in the MOGAD population [35].

## Use of AI tools declaration

The authors declare they have not used Artificial Intelligence (AI) tools in the creation of this article.

## References

[1] Hor JY, Fujihara K (2023). Epidemiology of myelin oligodendrocyte glycoprotein antibody-associated disease: a review of prevalence and incidence worldwide. Front Neurol.

[2] Banwell B, Bennett JL, Marignier R (2023). Diagnosis of myelin oligodendrocyte glycoprotein antibody-associated disease: International MOGAD Panel proposed criteria. Lancet Neurol.

[3] Fadda G, Flanagan EP, Cacciaguerra L (2022). Myelitis features and outcomes in CNS demyelinating disorders: comparison between multiple sclerosis, MOGAD, and AQP4-IgG-positive NMOSD. Front Neurol.

[4] Uzawa A, Oertel FC, Mori M (2024). NMOSD and MOGAD: an evolving disease spectrum. Nat Rev Neurol.

[5] Schoeps VA, Virupakshaiah A, Waubant E (2024). Unveiling the Performance of Proposed MOGAD Diagnostic Criteria. Neurology.

[6] Bartels F, Lu A, Oertel FC (2021). Clinical and neuroimaging findings in MOGAD–MRI and OCT. Clin Exp Immunol.

[7] Perez-Giraldo G, Caldito NG, Grebenciucova E (2023). Transverse myelitis in myelin oligodendrocyte glycoprotein antibody-associated disease. Front Neurol.

[8] Ciron J, Cobo-Calvo A, Audoin B (2020). Frequency and characteristics of short versus longitudinally extensive myelitis in adults with MOG antibodies: a retrospective multicentric study. Mult Scler.

[9] Sun X, Liu M, Luo X (2022). Clinical characteristics and prognosis of pediatric myelin oligodendrocyte glycoprotein antibody-associated diseases in China. BMC Pediatr.

[10] Armangue T, Olivé-Cirera G, Martínez-Hernandez E (2020). Associations of paediatric demyelinating and encephalitic syndromes with myelin oligodendrocyte glycoprotein antibodies: a multicentre observational study. Lancet Neurol.

[11] Satukijchai C, Mariano R, Messina S (2022). Factors associated with relapse and treatment of myelin oligodendrocyte glycoprotein antibody–associated disease in the United Kingdom. JAMA Netw Open.

[12] Wolf AB, Palace J, Bennett JL (2023). Emerging principles for treating myelin oligodendrocyte glycoprotein antibody-associated disease (MOGAD). Curr Treat Option Ne.

[13] Lai QL, Zhang YX, Cai MT (2021). Efficacy and safety of immunosuppressive therapy in myelin oligodendrocyte glycoprotein antibody-associated disease: a systematic review and meta-analysis. Ther Adv Neurol Disord.

[14] Lerch M, Bauer A, Reindl M (2023). The Potential Pathogenicity of Myelin Oligodendrocyte Glycoprotein Antibodies in the Optic Pathway. J Neuroophthalmol.

[15] Gontika MP, Anagnostouli MC (2018). Anti-Myelin Oligodendrocyte Glycoprotein and Human Leukocyte Antigens as Markers in Pediatric and Adolescent Multiple Sclerosis: on Diagnosis, Clinical Phenotypes, and Therapeutic Responses. Mult Scler Int.

[16] Weber J, Bernsdorff N, Robinson T (2021). Diagnosis of multiple sclerosis in times of MOG and AQP4 autoantibody testing - A monocentric study. J Neurol Sci.

[17] Sheppard M, Laskou F, Stapleton PP (2017). Tocilizumab (actemra). Hum Vaccin Immunother.

[18] Schirmer M, Muratore F, Salvarani C (2018). Tocilizumab for the treatment of giant cell arteritis. Expert Rev Clin Immunol.

[19] Si S, Teachey DT (2020). Spotlight on tocilizumab in the treatment of CAR-T-cell-induced cytokine release syndrome: clinical evidence to date. Ther Clin Risk Manag.

[20] Heo YA (2020). Satralizumab: First Approval. Drugs.

[21] Zhang C, Zhang M, Qiu W (2020). Safety and efficacy of tocilizumab versus azathioprine in highly relapsing neuromyelitis optica spectrum disorder (TANGO): an open-label, multicentre, randomised, phase 2 trial. Lancet Neurol.

[22] Sbragia E, Boffa G, Varaldo R (2024). An aggressive form of MOGAD treated with aHSCT: A case report. Mult Scler.

[23] Masuccio FG, Lo Re M, Bertolotto A (2020). Benign SARS-CoV-2 infection in MOG-antibodies associated disorder during tocilizumab treatment. Mult Scler Relat Disord.

[24] McLendon LA, Gambrah-Lyles C, Viaene A (2023). Dramatic Response to Anti-IL-6 Receptor Therapy in Children With Life-Threatening Myelin Oligodendrocyte Glycoprotein-Associated Disease. Neurol Neuroimmunol Neuroinflamm.

[25] Kwon O, Choi J (2024). Effective Prevention with Maintenance Treatment of Tocilizumab in Pediatric MOG-IgG Associated Disorder (MOGAD) Relapse. Ann Child Neurol.

[26] Kang YR, Kim KH, Hyun JW (2024). Efficacy of tocilizumab in highly relapsing MOGAD with an inadequate response to intravenous immunoglobulin therapy: A case series. Mult Scler Relat Disord.

[27] Elsbernd PM, Hoffman WR, Carter JL (2021). Interleukin-6 inhibition with tocilizumab for relapsing MOG-IgG associated disorder (MOGAD): A case-series and review. Mult Scler Relat Disord.

[28] Kim S, Kim S, Jang Y (2023). Leptomeningeal Enhancement, a Phenotype of Myelin Oligodendrocyte Glycoprotein Antibody-Associated Disease With Caudate Nucleus Involvement: A Case Report and Literature Review. J Clin Neurol.

[29] Virupakshaiah A, Moseley CE, Elicegui S (2024). Life-Threatening MOG Antibody-Associated Hemorrhagic ADEM With Elevated CSF IL-6. Neurol Neuroimmunol Neuroinflamm.

[30] Lee Y, Ahn SJ, Lee HS (2023). Myelin oligodendrocyte glycoprotein antibody-associated encephalitis after severe acute respiratory syndrome coronavirus 2 infection: a case report and retrospective case reviews. Encephalitis.

[31] Nagahata K, Suzuki S, Yokochi R (2022). Recurrent Optic Perineuritis With Myelin Oligodendrocyte Glycoprotein Antibody-Associated Disease Complicated With Granulomatous Polyangiitis. Cureus.

[32] Smoot K, Chen C, Cohan S (2021). Recurrent relapse after 20 years in a patient with MOG antibody disease: A case report. Neuroimmunol Rep.

[33] Kroenke E, Ankar A, Malani Shukla N (2022). Refractory MOG-Associated Demyelinating Disease in a Pediatric Patient. Child Neurol Open.

[34] Novi G, Gastaldi M, Franciotta D (2019). Tocilizumab in MOG-antibody spectrum disorder: a case report. Mult Scler Relat Disord.

[35] Schirò G, Iacono S, Salemi G (2024). The pharmacological management of myelin oligodendrocyte glycoprotein-immunoglobulin G associated disease (MOGAD): an update of the literature. Expert Rev Neurother.

[36] Hayward-Koennecke H, Reindl M, Martin R (2019). Tocilizumab treatment in severe recurrent anti-MOG-associated optic neuritis. Neurology.

[37] Dayrit KC, Chua-Ley EO (2024). Use of Tocilizumab Followed by Rituximab Desensitization on Relapsing Myelin Oligodendrocyte Antibody Disease: A Case Report. Cureus.

[38] Bilodeau PA, Vishnevetsky A, Molazadeh N (2024). Effectiveness of immunotherapies in relapsing myelin oligodendrocyte glycoprotein antibody-associated disease. Mult Scler.

[39] Lotan I, Charlson RW, Ryerson LZ (2020). Effectiveness of subcutaneous tocilizumab in neuromyelitis optica spectrum disorders. Mult Scler Relat Disord.

[40] Rempe T, Rodriguez E, Elfasi A (2024). Frequency, characteristics, predictors and treatment of relapsing myelin oligodendrocyte glycoprotein antibody–associated disease (MOGAD). Mult Scler Relat Disord.

[41] Ringelstein M, Ayzenberg I, Lindenblatt G (2021). Interleukin-6 receptor blockade in treatment-refractory MOG-IgG–associated disease and neuromyelitis optica spectrum disorders. Neurol Neuroimmunol Neuroinflamm.

[42] Guzmán J, Vera F, Soler B (2023). Myelin Oligodendrocyte Glycoprotein Antibody-Associated Disease (MOGAD) in Chile: lessons learned from challenging cases. Mult Scler Relat Disord.

[43] Rigal J, Pugnet G, Ciron J (2020). Off-label use of tocilizumab in neuromyelitis optica spectrum disorders and MOG-antibody-associated diseases: A case-series. Mult Scler Relat Disord.

[44] Powers JH, Mooneyham GC (2020). Psychiatric Symptoms in Pediatric Patients With Myelin-Oligodendrocyte-Glycoprotein-Immunoglobulin G-Antibody Positive Autoimmune Encephalitis: A Case Series. Psychosomatics.

[45] Jelcic I, Hanson JV, Lukas S (2019). Unfavorable structural and functional outcomes in myelin oligodendrocyte glycoprotein antibody–associated optic neuritis. J Neuroophthalmol.

[46] Hutto SK, Cavanagh JJ (2025). Advances in Diagnosis and Management of Atypical Demyelinating Diseases. Med Clin North Am.

[47] Schirò G, Iacono S, Andolina M (2023). Tocilizumab treatment in MOGAD: a case report and literature review. Neurol Sci.

[48] Schett G (2018). Physiological effects of modulating the interleukin-6 axis. Rheumatology (Oxford).

[49] Yoshida Y, Tanaka T (2014). Interleukin 6 and rheumatoid arthritis. Biomed Res Int.

[50] Tackey E, Lipsky PE, Illei GG (2004). Rationale for interleukin-6 blockade in systemic lupus erythematosus. Lupus.

[51] Koutsouraki E, Hatzifilipou E, Michmizos D (2011). Increase in interleukin-6 levels is related to depressive phenomena in the acute (relapsing) phase of multiple sclerosis. J Neuropsychiatry Clin Neurosci.

[52] Schneider A, Long SA, Cerosaletti K (2013). In active relapsing-remitting multiple sclerosis, effector T cell resistance to adaptive T(regs) involves IL-6-mediated signaling. Sci Transl Med.

[53] Fujihara K, Bennett JL, de Seze J (2020). Interleukin-6 in neuromyelitis optica spectrum disorder pathophysiology. Neurol Neuroimmunol Neuroinflamm.

[54] Takeshita Y, Fujikawa S, Serizawa K (2021). New BBB Model Reveals That IL-6 Blockade Suppressed the BBB Disorder, Preventing Onset of NMOSD. Neurol Neuroimmunol Neuroinflamm.

[55] Haramati A, Rechtman A, Zveik O (2022). IL-6 as a marker for NMOSD disease activity. J Neuroimmunol.

[56] Corbali O, Chitnis T (2023). Pathophysiology of myelin oligodendrocyte glycoprotein antibody disease. Front Neurol.

[57] Yao M, Wang W, Sun J (2024). The landscape of PBMCs in AQP4-IgG seropositive NMOSD and MOGAD, assessed by high dimensional mass cytometry. CNS Neurosci Ther.

[58] Horellou P, de Chalus A, Giorgi L (2021). Regulatory T Cells Increase After rh-MOG Stimulation in Non-Relapsing but Decrease in Relapsing MOG Antibody-Associated Disease at Onset in Children. Front Immunol.

[59] Moseley CE, Virupakshaiah A, Forsthuber TG (2024). MOG CNS Autoimmunity and MOGAD. Neurol Neuroimmunol Neuroinflamm.

[60] Whittam DH, Karthikeayan V, Gibbons E (2020). Treatment of MOG antibody associated disorders: results of an international survey. J Neurol.

[61] Nepal G, Kharel S, Coghlan MA (2022). Safety and efficacy of rituximab for relapse prevention in myelin oligodendrocyte glycoprotein immunoglobulin G (MOG-IgG)-associated disorders (MOGAD): A systematic review and meta-analysis. J Neuroimmunol.

[62] Yong KP, Kim HJ (2021). Demystifying MOGAD and Double Seronegative NMOSD Further With IL-6 Blockade. Neurol Neuroimmunol Neuroinflamm.

[63] Cochrane Library (2024). Safety and Efficacy of Tocilizumab in Patients With Myelin Oligodendrocyte Glycoprotein Antibody-associated Disease, NCT06452537.

[64] Cochrane Library (2022). A Study to Evaluate the Efficacy, Safety, Pharmacokinetics, and Pharmacodynamics of Satralizumab in Patients With Myelin Oligodendrocyte Glycoprotein Antibody-Associated Disease, NCT05271409.

[65] Kothur K, Wienholt L, Tantsis EM (2016). B Cell, Th17, and Neutrophil Related Cerebrospinal Fluid Cytokine/Chemokines Are Elevated in MOG Antibody Associated Demyelination. PLoS One.

